# Integrated Multi-Assay Culture Model for Stem Cell Chondrogenic Differentiation

**DOI:** 10.3390/ijms20040951

**Published:** 2019-02-22

**Authors:** Amy Prosser, Colin Scotchford, George Roberts, David Grant, Virginie Sottile

**Affiliations:** 1 Wolfson STEM Centre, School of Medicine, University of Nottingham, Nottingham NG7 2RD, UK; amy.prosser@nottingham.ac.uk; 2 Advanced Biomaterials Research Group, Faculty of Engineering, University of Nottingham, Nottingham NG7 2RD, UK; colin.scotchford@nottingham.ac.uk (C.S.); george.roberts@nottingham.ac.uk (G.R.); david.grant@nottingham.ac.uk (D.G.); 3 Arthritis Research UK Pain Centre, University of Nottingham, Nottingham NG7 2RD, UK

**Keywords:** stem cell differentiation, 3D culture, chondrogenesis, multimodal analysis, quantitative assay

## Abstract

Recent osteochondral repair strategies highlight the promise of mesenchymal progenitors, an accessible stem cell source with osteogenic and chondrogenic potential, used in conjunction with biomaterials for tissue engineering. For this, regenerative medicine approaches require robust models to ensure selected cell populations can generate the desired cell type in a reproducible and measurable manner. Techniques for in vitro chondrogenic differentiation are well-established but largely qualitative, relying on sample staining and imaging. To facilitate the in vitro screening of pro-chondrogenic treatments, a 3D micropellet culture combined with three quantitative GAG assays has been developed, with a fourth parallel assay measuring sample content to enable normalisation. The effect of transforming growth factor beta (TGF-β) used to validate this culture format produced a measurable increase in proteoglycan production in the parallel assays, in both 2D and 3D culture configurations. When compared to traditional micropellets, the monolayer format appeared less able to detect changes in cell differentiation, however in-well 3D cultures displayed a significant differential response. Effects on collagen 2 expression confirmed these observations. Based on these results, a microplate format was optimised for 3D culture, in a high-throughput in-well configuration. This model showed improved sensitivity and confirmed the 3D micropellet in-well quantitative assays as an effective differentiation format compatible with streamlined, high-throughput chondrogenic screens.

## 1. Introduction

Mesenchymal stem cells (MSCs) are a multipotent cell type able to differentiate into osteoblasts, chondrocytes or adipocytes [1,2,3,4]. Differentiation of MSCs along osteogenic and chondrogenic lineages can be recapitulated with established protocols [5,6,7], and analysed through staining of cells or sections [8]. Whilst these analytical methods can provide generic histology information, they do not provide quantitative measurements of cell differentiation, which would be desirable for treatment screening especially if they could be performed on multiple samples analysed in parallel.

Chondrogenic differentiation can be induced in MSC cultures by treating with a serum-free medium containing dexamethasone, ascorbic acid, l-proline, insulin, selenous acid, transferrin, and sodium pyruvate [7,9], further supplemented with members of the transforming growth factor beta (TGF-β) family. In vitro chondrogenic differentiation is customarily carried out for three weeks using 3D micromass culture [7], but more recent studies have also introduced chondrogenesis in 2D monolayer format [10]. Differentiation of MSCs in response to pro-chondrogenic treatment is characterised by an increased synthesis of collagens, proteoglycans, and glycosaminoglycans (GAGs), which can be detected using histological stains such as Safranin-O, Alcian Blue, or DMMB (1,9-dimethylmethylene blue) [11,12]. Additional chondrogenic readouts include visualisation and quantification of collagen II and X using immunohistochemistry, western blotting, and RT-PCR [5,7,8]. These methods involve significant sample processing, and are not easily amenable to high throughput quantitation, unlike some existing assays available for osteogenic and adipogenic differentiation. Alkaline phosphatase activity and alizarin red staining, two common techniques used to study osteogenesis, offer quantitative protocols compatible with a high-throughput 96-well plate format [6,13]. Similarly, adipogenic differentiation is amenable to multiwell plate measurements [14]. For in vitro chondrogenesis however, current methods typically involve cell differentiation as micromass pellets in individual 15 mL falcon tubes, followed by time-consuming pellet sectioning and staining to confirm differentiation in a qualitative rather than quantitative capacity.

MSCs represent a promising cell source for the treatment of osteochondral defects [15,16,17], and translating their potential to the clinic requires quantitative differentiation and analytical techniques to develop robust methods for large scale investigations. Several studies have attempted to address the limitations of standard 3D chondrogenic culture conditions, and adapted the traditional technique from a 15 mL polypropylene tube to a 96-well plate format [18]. However, a rapid assay allowing GAG quantification in each well is not yet available, as recent adaptations of Alcian Blue and Safranin-O based stains still require the use of an intermediate dot blotting step to enable GAG quantification [18,19]. To overcome these enduring limitations, this study has sought to develop a high throughput 3D differentiation method in multiwell plates through the evaluation of 2D and 3D cultures. Based on this optimised culture protocol, a novel analytical model combining three differentiation assays for chondrogenesis has been established for rapid, quantitative, and in-well measurement of GAG production.

## 2. Results

### 2.1. Optimising In Vitro Conditions for MSC Chondrogenesis

**Figure 1:**
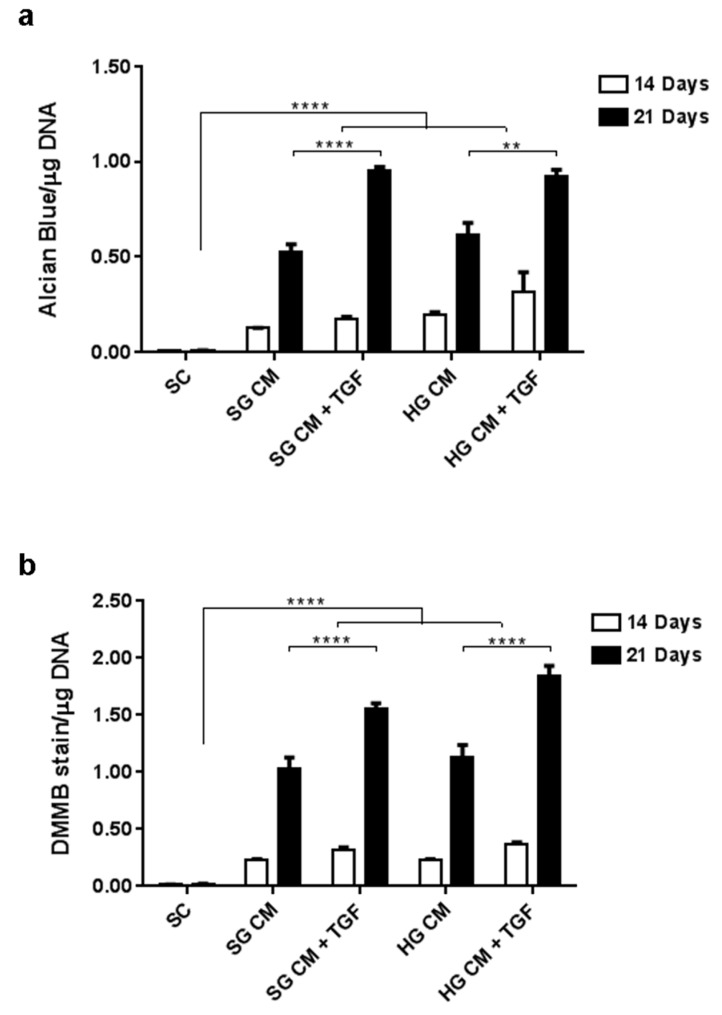
Comparison of standard glucose (SG) and high glucose (HG) media formulations on MSC chondrogenic differentiation assessed in 3D culture at day 14 and 21. (**a**) Alcian blue assay. (**b**) DMMB assay (*n* = 6). CM: chondrogenic medium, CM + TGF: chondrogenic medium supplemented with TGF-β1. ** *p* < 0.01, **** *p* < 0.0001. SC: Standard Culture medium, CM: chondrogenic differentiation medium, SG: standard glucose, HG: high glucose.

To define optimal conditions in support of chondrogenic differentiation in vitro, the efficacy of standard glucose (SG, 1 g/L) and high glucose (HG, 4.5 g/L) containing media, which have both been reported for chondrogenic differentiation protocols [20,21], was compared using in vitro assays. Cell response was analysed in mutliwell plates using micropellet cultures and analysed after 14 and 21 days, using the Alcian Blue and DMMB assays (Figure 1). Measurements of glycosaminoglycans (GAGs) produced were carried out using the established DMMB assay, and an assay based on Alcian blue, a widely used dye for chondrogenic differentiation [19,22]. The original staining protocol [19] was modified to allow direct quantification of GAG production in culture wells, without the need for membrane blotting.

Glucose concentration did not appear to significantly alter Alcian Blue or DMMB assay results after 3D differentiation for up to 21 days (*p* > 0.05). In this micropellet model however, both SG and HG media showed significantly higher GAG production in the presence of TGF-β, with a 76% and 50% increase in Alcian Blue staining and a 51% and 64% increase in DMMB staining for SG and HG respectively. HG-based CM medium was observed to enhance pellet formation and compaction in the initial stage of chondrogenic culture, which led to the production of larger, rounder pellets seemingly more robust and stable. By contrast, wells treated with SG-based CM contained several small cell aggregations rather than the larger single pellets present in HG-based CM cultures. Consequently, all CM conditions for subsequent experiments adopted high glucose DMEM since this did not significantly alter the GAG production and facilitated easier handling and more robust pellet production.

### 2.2. Comparing 2D and 3D Chondrogenic Differentiation Models

**Figure 2:**
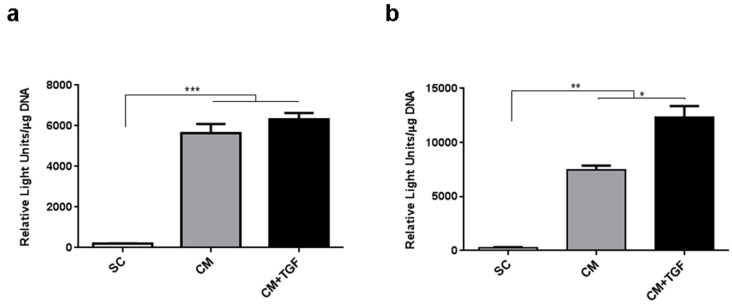
Collagen 2 reporter activity in MSC cells following 14-day chondrogenic treatment in either 2D monolayer (**a**) or 3D micromass format (**b**). Error bars represent the standard error of the mean (*n* = 3). * *p* < 0.05, ** *p* < 0.01, *** *p* < 0.001. SC: Standard Culture medium, CM: Chondrogenic differentiation medium, CM + TGF: chondrogenic medium supplemented with TGF-β1.

Since monolayer differentiation as tested in the first experiment did not provide clear discrimination between different chondrogenic conditions, a direct comparison between 2D monolayer differentiation and 3D pellet culture was performed to investigate whether the 3D chondrogenesis format would deliver greater sensitivity. Collagen type II (Coll2) is one of the first cartilage specific matrix proteins to be expressed when cells become committed to the chondrogenic lineage [23]. To further evaluate the MSCs response in different chondrogenesis models, a reporter construct enabling luciferase expression under control of the Col2α1 promoter was used for quantitative assays [24]. 2D monolayer and 3D pellet culture formats were compared over 14 days and reporter activity was measured in the presence or absence of TGF-β1 supplementation (Figure 2).

In both culture formats, CM treatment was found to significantly increase the relative Coll2 promoter activity compared to the SC control. However, the addition of TGF-β1 in monolayer culture did not result in a significant further increase compared to CM alone (Figure 2a), while its addition to the 3D micromass culture model led to a further significant increase compared to 3D CM cultures (Figure 2b). Comparing the response observed in the 3D micromass culture format and monolayer cultures suggested that 2D culture would not provide sufficient sensitivity to precisely evaluate chondrogenic response in MSCs.

**Figure 3:**
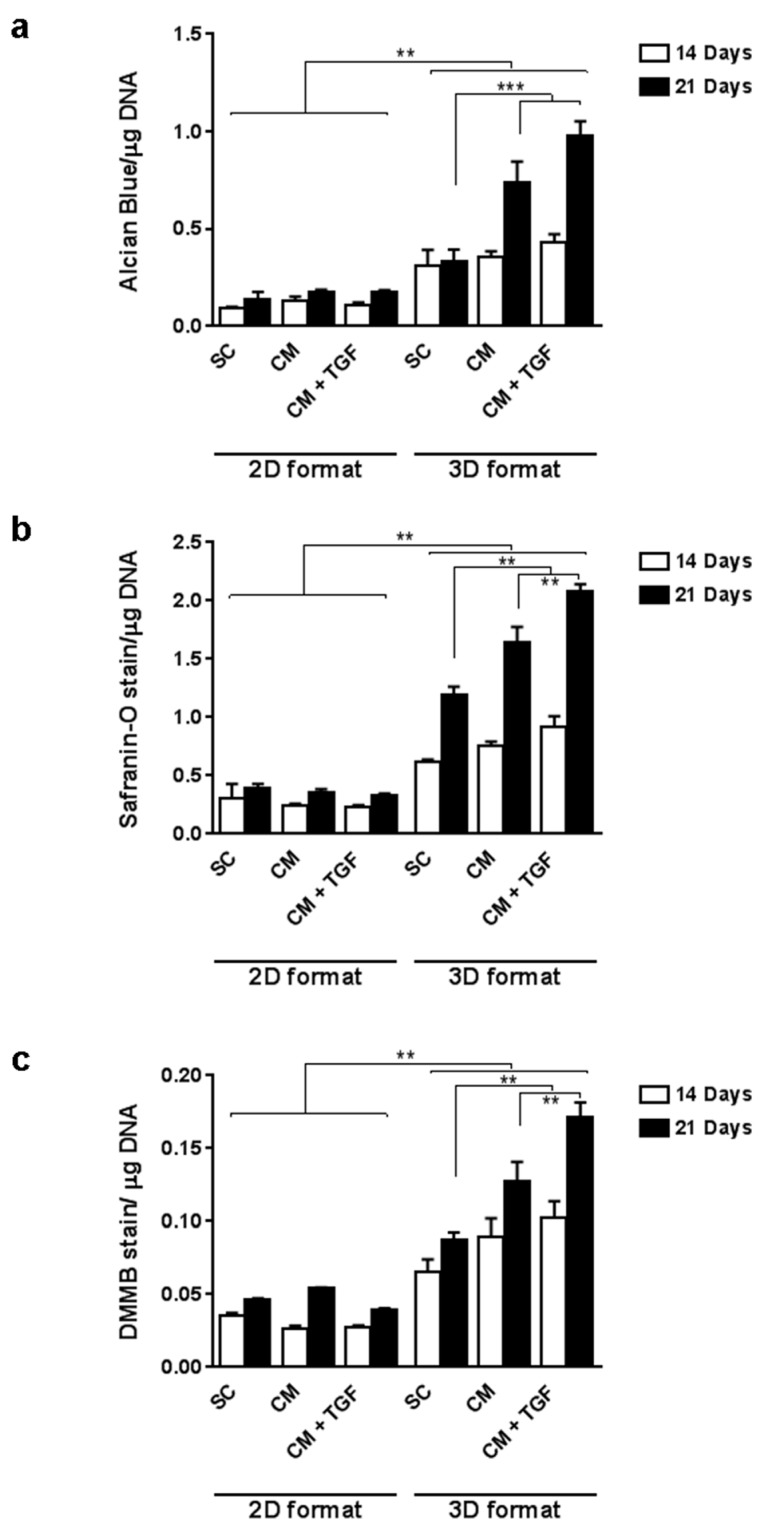
MSC chondrogenic response in 2D monolayer culture and 3D micropellet culture assessed by Alcian Blue assay (**a**, *n* = 4), Safranin-O assay (**b**, *n* = 4) and DMMB assay (**c**, *n* = 8). Error bars represent the standard error of the mean. ** *p* < 0.01, *** *p* < 0.001. SC: standard culture medium, CM: chondrogenic differentiation medium, CM + TGF: chondrogenic medium supplemented with TGF-β1.

To develop a mutliwell chondrogenic assay harnessing the benefits of 3D culture, micropellets were prepared by cell aggregation in V-shaped 96-well plates, and compared to 2D monolayer cultures after 14 and 21 days of treatment. In order to increase the accuracy of the quantitative analysis, an additional proteoglycan measurement parameter based on the quantitation of Safranin-O staining was developed to complement the Alcian Blue and DMMB assays. The triple differentiation assay was performed using Alcian Blue, Safranin-O, and DMMB in parallel to measure GAGs in monolayer (2D) and micropellet (3D) formats (Figure 3). All three assays indicated a significant difference between monolayer cultures and pellet cultures across all conditions at both time points, and the overall trend from the three assays was consistent with the increase in staining observed between day 14 and day 21. Monolayer cultures did not present significant increases in Alcian Blue (Figure 3a) or Safranin O (Figure 3b) staining in CM compared to standard conditions (SC) at either time point. Similarly, monolayer cultures analysed with DMMB staining (Figure 3c) did not significantly differ with time or media composition (*p* > 0.05). Assays run on 3D culture samples, in contrast, did show a significant difference between the SC control and CM-based media by day 21. Safranin-O and DMMB assays showed a further significant increase upon addition of TGF-β1 in 3D micropellet cultures after 21 days.

### 2.3. Comparing the Standard 3D Tube and the Micropellet Format for Chondrogenic Differentiation

**Figure 4:**
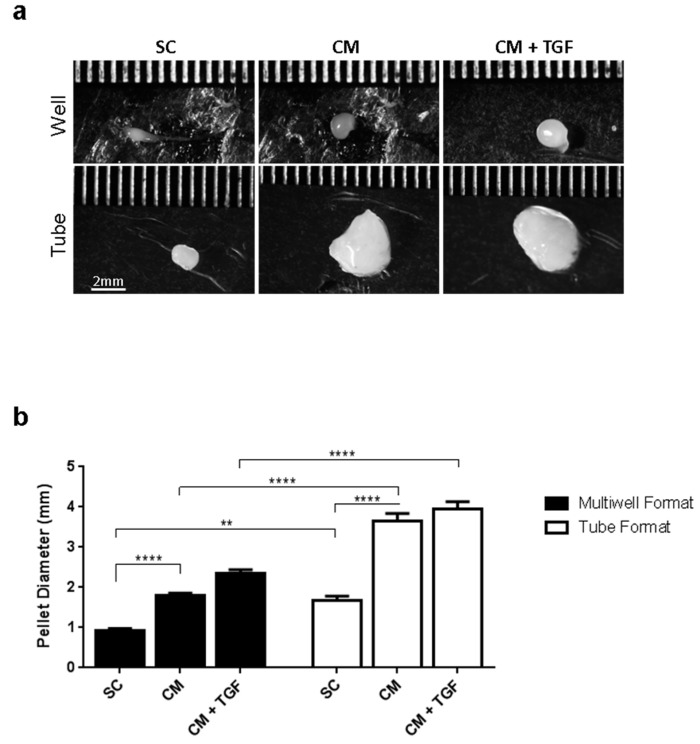
Analysis of chondrogenic pellets after 21 days culture using the micropellet plate method (blue) or the 15 mL falcon method (red). (**a**) Microscopic examination. (**b**) Size of chondrogenic pellets cultured measured along the diameter. The distance between the linear ruler marks is 0.5 mm. ** *p* < 0.01, **** *p* < 0.0001 (*n* = 4). SC: standard culture medium, CM: chondrogenic differentiation medium, CM + TGF: chondrogenic medium supplemented with TGF-β1.

**Figure 5:**
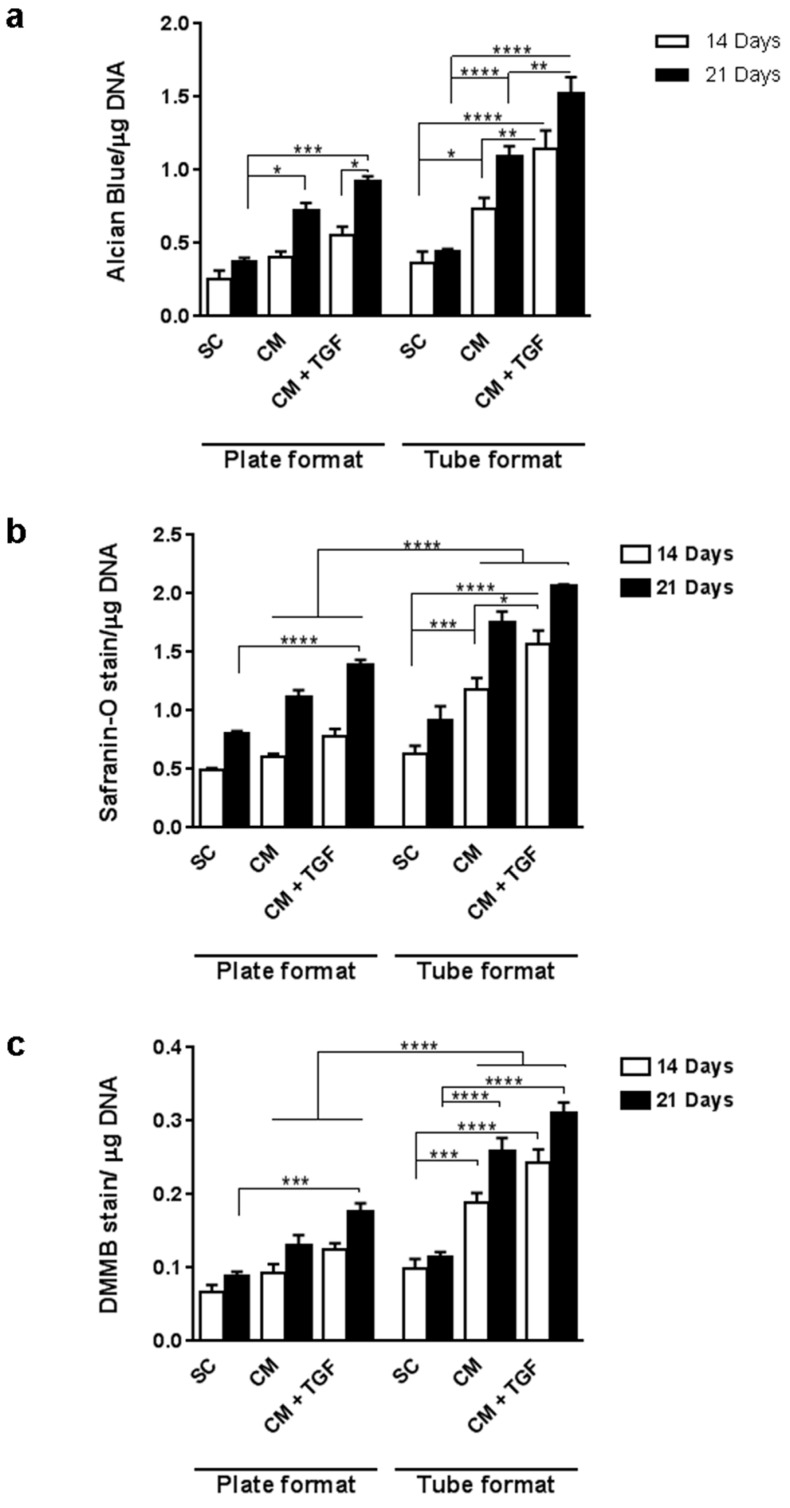
Comparison of MSC response to chondrogenic conditions analysed in 3D using micropellets (V-shape 96-well plates) and 15 mL falcon tubes. Cultures were analysed by Alcian Blue ((**a**), *n* = 4), Safranin-O ((**b**), *n* = 4) and DMMB ((**c**), *n* = 8) assays at 14 and 21 days. * *p* < 0.05, ** *p* < 0.01, *** *p* < 0.001, **** *p* < 0.0001. SC: standard culture medium, CM: chondrogenic differentiation medium, CM + TGF: chondrogenic medium supplemented with TGF-β1.

The two different 3D chondrogenesis culture formats were investigated in parallel to compare their degree of sensitivity for chondrogenic screening. Micropellet culture in conical multiwell plates was compared to the 15 mL falcon tube standard method. Resulting cell pellets were imaged after 21 days for size and shape comparison (Figure 4), and quantitatively analysed after 14 and 21 days using the triple Alcian Blue, Safranin-O, and DMMB assay (Figure 5).

Pellets imaged in Figure 4a were representative of the biological replicates produced, although the marked variation of the SC pellets in 15 mL tubes could not be accurately recorded, as approximately half of these disintegrated into smaller cell aggregates spread around the bottom surface of the tube. Pellet diameter measurements indicated that the falcon tube method produced significantly larger pellets than those cultured using the 96-well plate method (Figure 4b), which could be expected as they were initially seeded with 10 times more cells (10^6^ cells/pellet in 15 mL tubes, 10^5^ cells/pellet in well plates).

Pellets were further analysed for GAG production using Alcian Blue, Safranin-O, and DMMB assays (Figure 5). All three assays demonstrated an increase in GAG production over time, as well as a significant increase between SC and CM conditions. The addition of TGF-β1 significantly enhanced Alcian Blue staining, and after 21 days a significant increase in staining was observed in all three assays with addition of TGF-β1. Differentiation performed in 15mL tubes followed the same overall trend as micropellets, with an increase in signal over time and a significant difference between SC and CM conditions. A significant increase in staining was observed between 14 and 21 days for all three assays in CM-based treatments, while GAG production in pellets cultured in SC medium did not significantly increase over time. Overall, the 15 mL tube method led to higher GAG production compared to the plate method at both time points and across all three media compositions.

**Figure 6:**
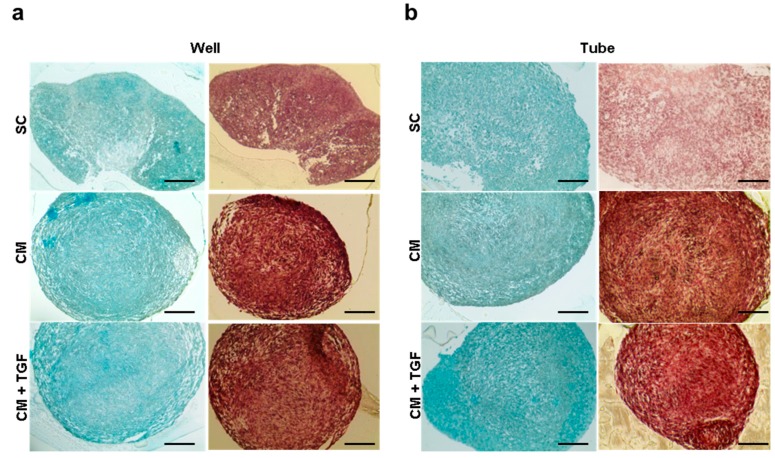
Micrographs of MSC chondrogenic pellet sections after 21 days of differentiation using the micropellet plate format (**a**) or 15 mL tube format (**b**), stained with Safranin-O (right column) and Alcian Blue (left column) Bar = 0.5 mm.

Pellet morphology and differentiation were imaged using Safranin-O and Alcian Blue staining on day 21 sectioned pellets (Figure 6). Similarly to the results obtained with the quantitative assays, the SC-based cultures showed poorly aggregated structures, possibly due to the presence of serum. Both CM-based differentiation formats produced pellets with a compacted capsule of cells around the edge and areas in the centre with a higher extracellular matrix to cell ratio indicative of chondrogenic maturation Samples obtained in the presence of TGF-β1 exhibited more homogenous staining across the pellet structures.

## 3. Discussion

TGF-β signalling plays an active role in regulating chondrocyte proliferation and differentiation, and the three TGF-β isoforms are expressed in the perichondrium of developing bones and in hypertrophic chondrocytes [25,26]. TGF-β1 in particular is routinely added to culture medium in vitro as it is known to promote chondrogenic differentiation from mesenchymal cells [7,9]. In this study, an in-well analysis approach was developed to enable streamlined multimodal quantitative analysis of MSC chondrogenic response in vitro. TGF-β1 response was used to optimise a 3D culture format for MSC differentiation and develop three quantitative and normalised multiwell assays to support large scale differentiation screens.

### 3.1. Chondrogenic Response in 2D vs. 3D Culture Formats

The standard chondrogenic differentiation format has traditionally relied on 3D micromass pellet cultures maintained using 15mL conical tubes [7]. This format is not easily amenable to scale-up and relatively low throughput for chondrogenic screening, and hence justify research into more efficient formats such as multiwell assays. While monolayer cultures would resolve these practical issues, they showed reduced ability to trigger collagen 2 expression during differentiation treatment, suggesting that the use of a multiwell plate format would require the retention of a 3D configuration. Collagen type II is one of the main cartilage-specific matrix proteins to be expressed when cells commit to the chondrogenic lineage [7,23]. When 3D micropellets were produced in 96-well plates, the chondrogenic response was observed after 21-day chondrogenic treatment and significantly enhanced with TGF-β1, in line with previous reports [27,28], confirming the suitability of this 3D plate format for chondrogenesis screening.

There have been some examples in the literature of chondrogenic studies in a monolayer culture format using embryonic stem cells [29], primary chondrocytes or marrow derived MSCs [30,31], to enable direct chondral morphology scoring. After 14 days of monolayer culture, the primary chondrocytes reportedly contained some cells with a hypertrophic chondroblast morphology; however, MSC cultures had fewer cells with a mature phenotype and produced lower overall chondral scores, with high ratio of cells to surrounding matrix, which suggested poor chondrogenic differentiation. Characterisation of the cellular response in both formats could be further refined using gene expression analyses such as qPCR to monitor the expression of other lineage markers.

### 3.2. 3D In-Well Micropellets for Chondrogenic Screening

Development of the direct in-well chondrogenic differentiation system in a V-shape plate format was compared with 3D pellet culture in 15 mL conical tubes, considered to be the traditional in vitro chondrogenesis format [7]. GAG production measured using Alcian Blue, Safranin-O, and DMMB staining was higher in 15mL tubes than in micropellets after both 14 and 21 days, with both methods producing a similar trend. After 21 days, both methods were equally sensitive and detected a significant enhancement in GAG levels in response to TGF-β1 addition, indicating their suitability to measure the effect of molecules and treatments on chondrogenesis. While the 15mL tube format was more sensitive at day 14 and could detect a significant TGF-β enhancement in GAG production, results obtained from the in-well micropellet cultures were only significant at the later time-point.

One major difference between the two 3D methods was the initial cell number seeded, and the resulting size of the pellet. The cell concentration of the starting suspension was kept constant, but the initial volumes required for the pellet set up were different. Consequently, pellets in the 15 mL tube method were initially seeded with 10^6^ cells each, whilst in-well micropellets were seeded with 10^5^ cells/well. Despite initially starting with ten times more cells than for the plate method, the 15 mL tube pellets only achieved between 3.8- and 7.6-times larger volumes after 21 days. The difference in initial cell number and pellet size may explain why the 15 mL tube method was more sensitive than the plate method at day 14, as the initial cell aggregates were larger in tubes, and thus may have required less cell proliferation before reaching sufficient aggregation to initiate differentiation. After a week of culture, pellets produced by the 15 mL tube method had rounded up to form almost spherical pellets, which did not substantially change in size throughout the rest of the culture period. Micropellets produced by the multiwell plate method also formed spherical pellets within a few days, but these continued to increase in size throughout the first two weeks of culture, before reaching a stage where no further net increase in size was observed. This suggested that at the initial stage of micropellet culture, cells may undergo more proliferation and less differentiation than the 15 mL tube method, which could explain the delay in chondrogenesis and reduced sensitivity at day 14.

Compared to seeding amounts used in other studies, the conditions used for the 15 mL culture method were in the higher the range, while those for the multiwell plate format were in the lower range. The original method [7] contained 2 × 10^5^ cells/pellet, and this concentration has been used in some subsequent studies [22], although others have used higher concentrations [21]. A study evaluating the optimum MSCs pellet size for chondrogenesis in the 15 mL tube format reported initial cell seeding numbers of 8 × 10^5^ cells/pellet to 1.6 × 10^6^ cells/pellet [32]. Increasing the initial seeding concentration in the multiwell plate format might however have an overall negative effect on chondrogenesis, as it would increase the risk of pellet acidification, which could in turn lead to pellet disaggregation. The maximum in-well volume capacity in a 96-well plate is 250 μL medium, whereas a conical tube can accommodate much larger volumes. Consequently, to avoid the risk of pellet acidification medium was replaced every day, whereas medium changes every 2–3 days were sufficient for the 15 mL tube format in agreement with other studies [7,21,22,32]. Less frequent medium changes, and thus fewer pellet disruptions, may also have contributed to more rapid pellet aggregation in the 15 mL tubes, possibly favouring earlier chondrogenesis onset which might lead to increased sensitivity at the earlier time point (day 14). However, this possible difference was transient, as the multiwell format showed comparable sensitivity by day 21.

Further optimisation of multiwell pellet culture could be obtained by comparing the efficiency of different well seeding densities for MSC chondrogenesis. The development of a medium perfusion system compatible with a multiwell culture format might also help minimise well acidification and maintain constant conditions for chondrogenesis [33,34] at higher seeding densities, although the effect of flow-perfusion on differentiation has been debated [35].

In addition to the difference in sensitivity observed at day 14 of culture, the two 3D culture formats tested here also produced diverging results for the pellet cultures maintained in SC media (negative controls). Whilst serum-containing medium has been shown to be detrimental to chondrogenic differentiation of MSCs [36], the micropellet plate method was able to sustain single aggregates throughout the 21-day period, while analysis of SC control pellets in the 15 mL tube method was challenging due to their fragmentation into smaller pellets dispersed around the walls at the bottom of the tube after the first few days of culture. These smaller pellets then failed to increase in size or compact to form robust pellets, with some of the replicates further disaggregating over time. The larger pellet size in the 15 mL tube format may have contributed to their poor survival in SC conditions, as cells may have experienced poorer nutrition and dilution of waste metabolic products through the larger aggregate structure, which coupled with the unfavourable serum presence, may have rendered larger SC pellets more susceptible to disaggregation. The in-well micropellet plate method, by contrast, was able to maintain single pellets in SC media, albeit with an altered morphology. These pellets consisted of a small spherical core with ‘tails’ bringing the overall shape closer to an elongated disc than a sphere. Despite this altered micropellet morphology, SC culture could be carried out over a 21-day period with samples that could be processed for analysis. For chondrogenic screening purposes, the ability to test possible active compounds in SC medium as seen in the multiwell plate method is advantageous, as this would allow assessment of their specific differentiation capacity in the absence of any other chondrogenic prompts.

### 3.3. Quantitative In-Well Assays for a High Throughput Differentiation Format

The 3D cell culture system established here allowed the in-well culture and subsequent measurement of chondrogenic differentiation using three quantitative GAG assays for treatment screening. Used mostly to stain tissue samples for imaging, Safranin-O staining of proteoglycans present in cartilage has also been used for a semi-quantitative approach involving loading of the samples onto a dot blot, dye extraction and then measurement of the eluted fraction [18,37], which could be both technically demanding and prone to intrinsic variability. Here, an alternative in-well method was developed to enable the culture and subsequent quantification of the GAGs directly into each culture well, without the need for membrane blotting or leaching of the samples. The integration of this novel Safranin-O protocol with Alcian blue and DMMB assays generated three comprehensive quantitative GAG assays which, when combined to DNA content analysis for normalisation, provided a robust yet practical methodology for large scale chondrogenic screening with minimal sample processing steps. The parallel establishment of this in-well combined quantitative assay with the development of a 3D in-well micropellet culture format provides a scalable and potentially automatable integrated approach to chondrogenic screening. Further developments will now be envisaged through the implementation of additional assays targeting matrix components, such as type I and X collagens, and immunophenotying approaches such as ELISA measurements. For multilineage differentiation assays, this in-well approach for chondrogenesis could also be run in parallel to Alizarin Red and Oil Red O staining measurements for osteogenesis and adipogenic assays, respectively.

The optimised 3D micropellet plate format demonstrated superior sensitivity and reliability compared to 2D plate methods. It also performed successfully alongside the traditional and more cumbersome micromass pellet culture in 15 mL polypropylene tubes, achieving comparable sensitivity over 21 days. These results support the use of this new in-well assay as an improved approach for the screening of small molecules and chondrogenic treatments in a multiwell culture format, better suited to high throughput approaches for regenerative medicine.

## 4. Materials and Methods

Reagents were purchased from ThermoFisher Scientific (UK) unless otherwise stated.

### 4.1. Cell Culture

Human bone marrow mesenchymal stem cells (MSCs) from healthy donors [12,38] were cultured in standard culture medium (SC) containing Dulbecco’s Modified Eagle Medium supplemented with 10% (*v*/*v*) foetal calf serum, 1% (*v*/*v*) l-Glutamine, 1% (*v*/*v*) non-essential amino acids (NEAA) and 1% (*v*/*v*) penicillin/streptomycin at 37 °C and 5% CO_2_. Cells were passaged with 0.05% trypsin/EDTA. Three different cell culture formats were used to analyse chondrogenic differentiation: 2D monolayer culture in well plates, 3D micropellet culture in V-shaped well plates, and 3D pellet culture in 15 mL polypropylene conical tubes.

For 2D monolayer culture, 100 μL of a 2 × 10^4^ cells/mL cell suspension were added to each well of flat bottom 96-well plates. After a 24-h attachment period the medium was then replaced with the treatment condition and changed three times per week throughout the cell culture period.

For 3D micropellet culture, 100 μL of a 10^6^ cells/mL cell suspension was added to each well of V-shaped 96-well plate (Greiner Bio-One, Stonehouse, UK). Plates were spun in a Sigma 3–16 k centrifuge at 500× *g* for 5 min to pellet the cells, and after 24 h the media was replaced with 250 μL of each treatment condition. 70% of culture media was replaced every day throughout the culture period to avoid disrupting the cell pellet and prevent pellet acidification.

For standard 3D micromass pellet culture, 15 mL polypropylene tubes were used as previously described [3,7]. Briefly, 1 mL of a 10^6^ cells/mL cell suspension was added to each tube before centrifugation at 500 g in a sigma 3–16 centrifuge to form a cell pellet. After 24 h the medium was replaced with 2 mL of each treatment condition. Culture medium was replaced three times a week throughout the differentiation period.

Chondrogenic media (CM) used in all differentiation experiments contained DMEM supplemented with 1% (*v*/*v*) l-glutamine, 1% (*v*/*v*) NEAA, 1% (*v*/*v*) penicillin/streptomycin, dexamethasone (0.1 μM, Sigma-Aldrich, Gillingham, UK), ascorbic acid phosphate (50 μM, Sigma-Aldrich), sodium pyruvate (1 mM), l-proline (40 μg/mL), and 1× Insulin-Transferrin-Selenium Supplement (ITS+, Sigma-Aldrich). Both standard glucose (1 g/L) and high glucose (4.5 g/L) DMEM were analysed in this project, but unless otherwise stated, CM contained high glucose DMEM. In some samples, TGF-β1 (10 ng/mL) was added to CM medium where indicated.

### 4.2. Alcian Blue Quantification of GAG Production

Cultures were rinsed twice with PBS and fixed in 4% (*w*/*v*) paraformaldehyde (PFA) (VWR, UK) at 4 °C. Each well was incubated with 100 μL of 1% (*w*/*v*) Alcian Blue 8-GX (Sigma Aldrich) in 0.1 M HCl (pH 1.0) at room temperature overnight. After 4 PBS washes, 100 μL PBS were added before imaging with a Nikon Eclipse TS100 inverted microscope. Bound Alcian Blue was extracted at room temperature using 100 μL 6 M guanidine HCl on a plate shaker for 2 h, and quantification was achieved using an Infinite 200 micro-plate reader (Tecan, Reading, UK) at 650 nm absorption. Results were normalised to DNA content obtained from replicate wells analysed using the QUANT-IT PicoGreen kit according to manufacturer’s instructions as a read-out for relative cell numbers. Briefly, cells were lysed by replacing culture medium with 100 μL of sterile water and subjecting the samples to three freeze–thaw cycles from −20 to 37 °C. 100 μL of PicoGreen solution was then added to each well and agitated in the dark for 5 min at room temperature. The fluorescence intensity was measured at 480 nm excitation and 520 nm emission using an Infinite 200 microplate reader (Tecan). A DNA standard curve generated with calf thymus DNA (Sigma-Aldrich) was used to determine the concentration of DNA in each sample.

### 4.3. 1,9-Dimethylmethylene Blue (DMMB) Assay of GAG Production

DMMB is a cationic dye which binds to sulphate and carboxylate groups within GAGs, producing a concentration dependent metachromatic change [39]. After the culture period, wells were washed twice with PBS and 100 μL of sterile water was added to each well. Samples were freeze–thawed three times and sulphated GAGs were dissociated from other glycoproteins using a papain digest. 100 μL of 1% (*w*/*v*) papain solution (Sigma-Aldrich) in papain buffer (0.1 M sodium phosphate, 0.005 M cysteine hydrochloride and 0.005 M EDTA, pH 6.5) were added to each well and incubated at 60 °C for 24 h. Triplicate 50 μL aliquots were transferred to a clean 96-well plate and combined with 50 μL of DMMB solution (0.03 M sodium formate (Sigma-Aldrich), 0.046 mM DMMB (Sigma-Aldrich), 0.005% *v*/*v* ethanol and 0.002% formic acid (Sigma-Aldrich)). 540 nm absorption was measured on an Infinite 200 micro-plate reader (Tecan). Results were normalised to the DNA content measured in replicate wells using the QUANT-IT PicoGreen kit.

### 4.4. Quantification of GAG Production Using Safranin-O

PFA-fixed cultures were rinsed twice with PBS and 100 μL of 0.02% Safranin-O (Sigma-Aldrich) in 50 mM sodium acetate (pH 4.8) were added to each well for 20 min at room temperature. After three PBS washes, the Safranin-O dye was extracted using 10% cetylpyridinium chloride (Sigma-Aldrich) for 20 min on a plate shaker at 37 °C. Quantification was achieved by reading the absorption of the extracted dye at 530 nm using an Infinite 200 micro-plate reader (Tecan). Results were normalised to the DNA content using the QUANT-IT PicoGreen assay.

### 4.5. Pellet Histology

Pellets were washed twice with PBS and fixed in 4% PFA, and imaged using the Leica MZ16F microscope and pellet diameters were quantified using the ImageJ analysis software before wax-embedding for histological assessment. 10 µm thick sections were stained with Mayer’s Haematoxylin (Sigma-Aldrich) for 5 min, rinsed and then immersed into either 0.1% (*w*/*v*) Safranin-O (Sigma-Aldrich) acidified with a few drops of glacial acetic acid, or 1% (*w*/*v*) Alcian blue 8-GX (Sigma-Aldrich) in 0.1 M HCl (pH 1.0) for 30 min before imaging using an EVOS XL Core microscope.

### 4.6. Quantification of Luciferase Expression

A human Col2α1 promoter reporter driving luciferase expression was kindly provided by Dr T. Saito and Dr K. Chiba [24,40], and used to stably transfect MSCs. Luciferase expression was measured using the Luciferase Assay System (Promega, Southampton, UK). Cell pellets were washed with PBS and 300 μL of 0.1 M K_2_HPO_4_ (pH 7.8, Sigma-Aldrich), 2 mM EDTA (Sigma-Aldrich) added to each tube before pellets were homogenised. 100 μL of each sample were then rapidly frozen on dry ice and brought back to room temperature in a 37 °C water bath. 300 μL of lysis buffer containing 1.25 mg/mL lysozyme and 2.5 mg/mL BSA were added at room temperature and incubated for 10 min before aliquots of 100 µL were transferred to a 96 well plate. Finally, 100 μL of Luciferase Assay Reagent were added to each well at room temperature and plates were immediately read using a 2 s measurement delay followed by a 10 s measurement read with an Infinite 200 micro-plate reader (Tecan).

### 4.7. Statistical Analysis

All graphs and statistical analysis were performed using GraphPad Prism 6.0. One-way ANOVA analysis was performed using a Tukey post hoc correction showing **** *p* < 0.0001, *** *p* < 0.001, ** *p* < 0.01, and * *p* < 0.05. Error bars on graphs show the standard error of the mean.

## Abbreviations

hMSC	human mesenchymal stem cells
DMMB	1,9-dimethylmethylene blue
GAG	glycosaminoglycans
